# Substrate Recognition Properties from an Intermediate Structural State of the UreA Transporter

**DOI:** 10.3390/ijms232416039

**Published:** 2022-12-16

**Authors:** Manuel Sanguinetti, Lucianna Helene Silva Santos, Juliette Dourron, Catalina Alamón, Juan Idiarte, Sotiris Amillis, Sergio Pantano, Ana Ramón

**Affiliations:** 1 Sección Bioquímica, Departamento de Biología Celular y Molecular, Facultad de Ciencias, Universidad de la República, Iguá 4225, Montevideo 11400, Uruguay; 2 Biomolecular Simulations Group, Institut Pasteur de Montevideo, Mataojo 2020, Montevideo 11400, Uruguay; 3 Neurodegeneration Laboratory, Institut Pasteur de Montevideo, Mataojo 2020, Montevideo 11400, Uruguay; 4 Columbia University Irving Medical Center, Columbia University, New York, NY 10032, USA; 5 Department of Biology, National and Kapodistrian University of Athens, Panepistimioupolis, 15784 Athens, Greece

**Keywords:** *Aspergillus nidulans*, urea transport, AlphaFold2, binding site, molecular docking

## Abstract

Through a combination of comparative modeling, site-directed and classical random mutagenesis approaches, we previously identified critical residues for binding, recognition, and translocation of urea, and its inhibition by 2-thiourea and acetamide in the *Aspergillus nidulans* urea transporter, UreA. To deepen the structural characterization of UreA, we employed the artificial intelligence (AI) based AlphaFold2 (AF2) program. In this analysis, the resulting AF2 models lacked inward- and outward-facing cavities, suggesting a structural intermediate state of UreA. Moreover, the orientation of the W82, W84, N279, and T282 side chains showed a large variability, which in the case of W82 and W84, may operate as a gating mechanism in the ligand pathway. To test this hypothesis non-conservative and conservative substitutions of these amino acids were introduced, and binding and transport assessed for urea and its toxic analogue 2-thiourea, as well as binding of the structural analogue acetamide. As a result, residues W82, W84, N279, and T282 were implicated in substrate identification, selection, and translocation. Using molecular docking with Autodock Vina with flexible side chains, we corroborated the AF2 theoretical intermediate model, showing a remarkable correlation between docking scores and experimental affinities determined in wild-type and UreA mutants. The combination of AI-based modeling with classical docking, validated by comprehensive mutational analysis at the binding region, would suggest an unforeseen option to determine structural level details on a challenging family of proteins.

## 1. Introduction

Bacteria, fungi and plants are able to use urea as the sole nitrogen source for the biosynthesis of nucleic acids, lipids and proteins. This substance occurs in nature as a product of animal, plant and microbial metabolism. Additionally, it accounts for more than 70% of the applied synthetic nitrogen fertilizer [1,2]. However, as for other fertilizers, its nitrogen use efficiency is low and leads to the utilization of excessive amounts, with detrimental effects in air and water quality. Hence, in order to improve the ability of plants to use nitrogen more efficiently, research efforts aiming to understand the uptake and usage mechanisms of the main fertilizer nitrogen sources is of great importance [1,3].

Urea is taken up by fungi and plants through specific membrane permeases that belong to a specialized subfamily of urea/H^+^ symporters within the sodium:solute symporter (SSS) superfamily [4], functionally and evolutionarily separated from urea transport channels operating in bacteria and animals [5,6,7,8]. Characterized members of this group include the fungal transporters from *Saccharomyces cerevisiae* (ScDUR3) [9], *Paxillus involutus* (PiDUR3) [10], *Aspergillus nidulans* (UreA) [4], *Candida albicans* (CaDUR3) [11], and the plant ones from *Arabidopsis thaliana* (AtDUR3) [12,13], *Oryza sativa* (OsDUR3) [14], and *Zea mays* (ZmDUR3) [13]. Of note, overexpression of OsDUR3 in *Arabidopsis thaliana* improved the growth on low urea concentrations with a marked increase in root urea-uptake [14], highlighting the importance of the study of this group of transporters in order to increase the nitrogen usage efficiency of this compound.

**Figure 1:**
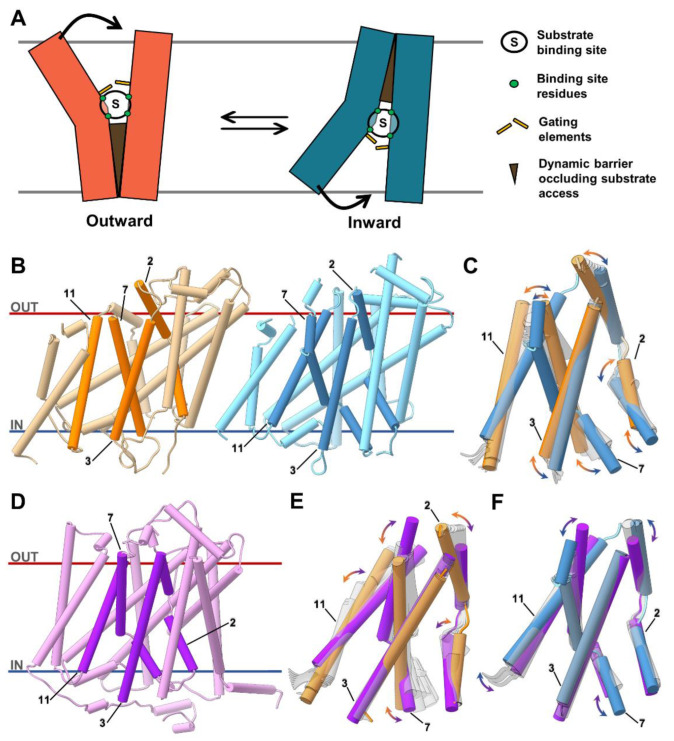
Comparison of the different UreA modeled conformations. (**A**) Schematic representation of the rocking-bundle mechanism where one domain (left) rocks against another less motile domain (right) to give access and release the substrate. Interface residues (green circles) from both domains form the substrate binding site, while substrate-triggered gating elements (yellow bars) can act as selectivity filters over or below the binding site. Specific and dynamic domains (brown triangle) operate as occlusion barriers to hinder substrate leakage. (**B**) Predicted outward-facing (orange) and inward-facing (blue) UreA structures with highlighted TMSs 2, 3, 7 and 11, respectively. (**C**) Comparison of outward- (orange) and inward-facing (blue) conformations of TMSs 2, 3, 7 and 11 that might compose the urea translocation pathway. Light gray structures are morphed (i.e., linearly interpolated) intermediates produced by UCSF Chimera’s morph conformations tool [37]. These frames indicate the possible movement in the TMSs when cycling between conformations. (**D**) One of the predicted UreA AF2 models (purple) with TMSs 2, 3, 7 and 11 (dark purple) highlighted. (**E**) Morphed intermediates (light gray structures) proposed that UreA’s AF2 (purple) and outward (orange) models have different TMSs 2 and 7 conformations. (**F**) Morphed intermediates (light gray) proposed a more modest mobility, but TMSs 2 and 7 were closer together in the AF2 model than in the inward model. Helices are shown as cylinders and less relevant protein loops were hidden to improve the visualization. An animation showing the continuous passage from one model to the other is provided in supplementary material to illustrate the differences between the different states.

The model fungus *A. nidulans* turned out to be an excellent model to study membrane transporters. Paradigmatic structure-function studies have allowed for unveiling relevant aspects of the biology of different membrane transporters [15]. In this way, we have focused on the study of UreA (UniProt Q5BGB2), the only high-affinity urea transport system in *A. nidulans* [4,16,17] as a representative of the fungi and plant urea/H^+^ symporters. UreA was isolated and named by Pateman et al. [18] more than 40 years ago to refer to mutants isolated as resistant to 2-thiourea and shown by transport assays and growth tests to be specifically impaired in urea transport. Note that UreA can also stand for one of the subunits of the bacterial and cyanobacterial urease (urea hydrolase) [19,20], while in *A. nidulans* urease subunits are named UreB, UreC and UreD [21]. This transporter, with a predicted structure composed of 15 α-helical transmembrane segments (TMS), transports urea as well as its toxic analogue 2-thiourea (2-TU). The transport of urea is inhibited by acetamide (ACM) [4]. A previous structure-function analysis of UreA was carried out, based on a dual approach involving both site-directed mutagenesis of aminoacidic residues conserved between UreA and characterized fungal and plant orthologs and random mutagenesis, combined with comparative modeling [16]. UreA was modeled in the two conformations known for this family: the closed “inward facing” conformation, modeled on the structure of the *Vibrio parehaemolyticus* sodium-glucose symporter vSGLT [22]; and the closed “outward facing” conformation based on the structure of the benzyl-hydantoin transporter, Mhp1, from *Microbacterium liquefaciens* [23]. Those structures are typically composed of two inverted five-helix bundles (the LeuT-fold), also found in other transporters with no sequence similarity and which do not share substrates [24]. Transporters with this structure have been proposed to work through a “rocking-bundle” mechanism, characterized by a substrate-induced alternation between the inward and outward-facing states, with concomitant opening and closing of specific gating elements (Figure 1A) [25,26,27,28]. Recently, computational and functional analysis have corroborated a ligand-driven conformational switching mechanism, from outward-open to inward-open conformations, for hSGLT1 [29] and vSGLT [30] transporters. For UreA, the structure-function analysis allowed for the identification of a number of residues, which, according to the model, are exposed to the solvent involved in substrate binding, recognition and translocation (namely, W82, Y106, A110, T133, D286, Y388 and Y437). Additionally, those amino acids lay in transmembrane helixes which, after the predicted model, coincide with those that in vSGLT bear key residues for substrate binding and translocation. Y106 and Y437 in particular, predicted to face each other in the inward facing conformation and to almost superimpose with residues of the vSGLT outside gate, were proposed to function as a selectivity filter [16].

In this work, we aim to deepen the structural characterization of UreA and its substrate-binding and selectivity residues by applying the newly introduced artificial intelligence (AI) based modeling approach AlphaFold2 (AF2) [31,32]. AF2′s de novo predictions are created using a deep learning algorithm that recognizes features from both experimentally determined structures and multiple sequence alignments (MSAs). Since its release, there have been efforts to use AF2 to explore alternative structures, especially for proteins featuring multiple states, including membrane transporters and receptors [33,34,35,36].

Unexpectedly, the AF2 prediction of UreA structure showed a model dissimilar to the closed inward- and outward-facing conformations previously described by comparative modeling, which could be considered an intermediate-like UreA conformation. To validate this structural prediction, we used molecular docking to explore the capability of this model to bind urea and its analogues 2-TU and ACM. To provide an exhaustive validation of theoretical results, we changed the physicochemical determinants of the putative binding site by site-directed mutagenesis and estimated the binding affinity by measuring urea uptake. We also explored the effect of variations in the chemical groups of the ligand by performing uptake experiments using 2-TU and ACM as well. Our work is an example of how combining AI-based methods with more “traditional” docking techniques, exhaustively validated by a molecular biology approach, can provide a way to further our knowledge on the structural characteristics of a difficult to approach family of transporter proteins.

## 2. Results

### 2.1. Characterization of a Theoretical Intermediate State of UreA and Its Binding Site

In Sanguinetti et al. (2014) [8], two closed-state UreA models comprising the transmembrane helices (TMSs) 2 to 13 were created using comparative modeling (Figure 1B) [16]. The models depicted UreA with inward- and outward-facing cavities based on the vSGLT of *V. parahaemolyticus* [22] and Mhp1 of *M. liquefaciens* [23] structures, respectively. These obtained models allowed pinpointing specific amino acids despite their low amino acid identity with the structurally characterized homologues [16]. The models suggested that critical residues in TMSs 2, 3, 7 and 11, correlate to substrate binding and recognition areas known to harbor key residues for substrate binding and translocation in vSGLT. Transporters of the SSS superfamily are thought to feature a “rocking bundle” mechanism in which inward- and outward-facing hydrophilic cavities can alternate (Figure 1C) [25,26,27,28]. This mechanism is thought to occur by substrate-induced effects that open and close specific gating amino acids. Thus, outward- and inward-facing conformations could be considered the beginning and end of urea transport through UreA. These conformations are characterized by higher vestibular volumes at the top of the TMSs 2, 3, 7 and 11 for the outward-facing cavity and at the bottom for the inward-facing one (Supplementary Figure S1A,B) and present RMSD of 3.9 Å between all TMSs.

In this work, we used the newly introduced AF2 approach to better understand structural differences between both states and the residues that determine the urea binding site. To that aim, we built an alternative UreA conformation using AF2 (Figure 1D). The top five AF2 models had comparable conformations, with higher variability found in the N- and C-terminal regions (Supplementary Figure S2) and a moderate-to-high AF2 confidence score (Supplementary Figure S3). Thus, the AF2 models have an overall moderate confidence in secondary structure element arrangement and amino acid locations. Comparison of UreA’s AF2 conformations with the outward- and the inward-facing models revealed an RMSD of 3.6 Å and 3.2 Å between the TMSs, respectively, with noticeable distances in the TMSs 2, 3, 7 and 11 (Figure 1E,F). AF2′s TMSs 2 and 7 were narrower when compared with both the outward- (Figure 1E) and inward-facing conformations (Figure 1F). AF2 conformations also proposed more restricted empty volumes between TMSs 2, 3, 7 and 11 than the others (Supplementary Figure S1C). Thus, the UreA model proposed by AF2 could be viewed as a structural intermediate between the inward and outward-facing conformations. These distinct models and modeling approaches are used here to gain new insights into the roles of important amino acids in specific structural states. However, despite a good correlation between the theoretical and experimental data (see below) the conformations showed here are not to be considered as high-resolution structures or representatives of UreA’s continuous translocation process.

**Figure 2:**
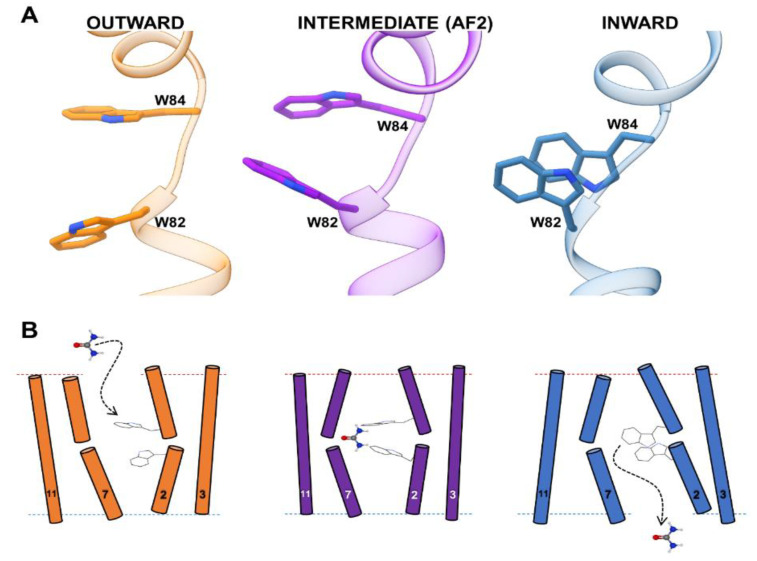
W82 and W84 orientations in the UreA conformations. (**A**) Comparison between the orientations of W82 and W84 in each predicted conformation. In the intermediate UreA state, these two residues facing each other form a potential groove that may stabilize urea in the putative binding site. For a video of the orientation transitions between conformations, see the supplemental material information. (**B**) A representation of the possible urea gating mechanism showing the potential flexibility of TMSs 2, 3, 7, and 11, W82, and W84.

Examination of TMSs 2 and 7 predicted by AF2 showed a large variability in the side chain orientations of W82, W84, N279, and T282 (Supplementary Figure S2). The indole rings of W82 and W84 at the broken TMS 2 can be more closely opposed to each other than in the outward- and inward-facing conformations (Figure 2A). Thus, these residues together could form a potential groove that may “sandwich” and stabilize urea, acting as a binding site. A similar mechanism was previously hypothesized for Y106 and Y437, also known to be involved in substrate selectivity [16]. In the inward-facing conformation, these two Tyr residues are facing each other and could act as a selectivity filter that “sandwich” urea, stabilizing the partial positive charge on the amide nitrogen through amide-pi stacking interactions. Moreover, TMS 7 seemed to move away from TMS2 in each conformation, suggesting some plasticity in the urea pathway (Supplementary Figure S4). TMS 7 residues N279 and T282 side chains also showed some orientational variability. However, this variability was less evident than in the case of W82 and W84. The change in the global arrangement of TMS 2 and TMS 7, together with the observed flexibility of the side chains of W82, W84, N279 and T282 in this putative binding site may suggest a urea-activated gating mechanism depicted in Figure 2B.

**Figure 3:**
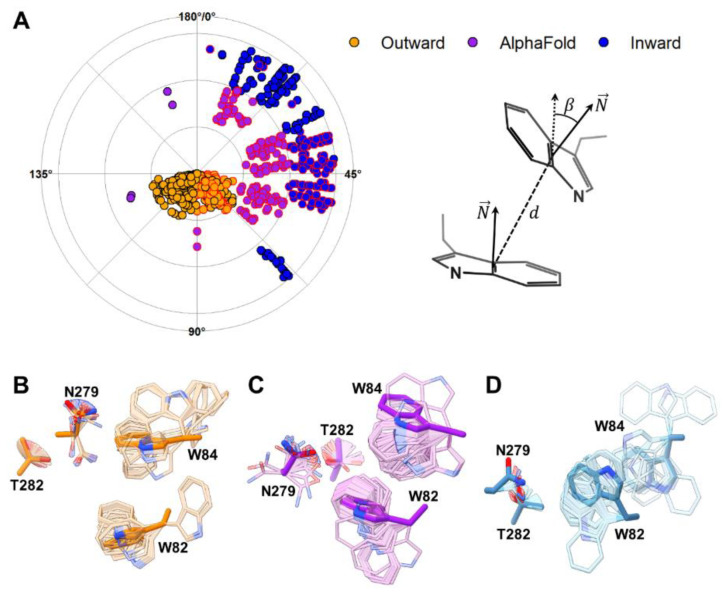
Docking of urea modulates the orientation of W82 and W84. (**A**) The radial plot of the angles between the planes defined by the indole rings of W82 and W84 and the schematic representation of angle (*β*) and distance (*d*) calculation. Red-bordered points show angles between 0° to 90° and distances up to 6 Å, thus, indicating that the sidechains could be potentially in an aromatic stacking orientation. Sampled angles are intercepted in all models between 45° and 67.5°, with the angles in the outward-facing model (**B**) reaching higher values than those sampled in the AlphaFold2 (**C**) and inward-facing (**D**) models.

To investigate this potential gating mechanism, we performed molecular docking of urea using Autodock Vina [38,39]. With a short runtime, this open-source program allows side chain flexibility sampling of a group of chosen residues. Docking of urea was performed considering the entire region between TMSs 2 and 7, leaving flexible the side chains of W82, W84, N279 and T282. This procedure resulted in a series of urea poses scattered throughout the pathway proposed in Figure 2B. In addition, side chain flexibility allowed sampling alternative angles between W82 and W84 (Supplementary Figure S5). The outward-facing model showed a higher concentration of poses near the outer side (top of the pathway, Supplementary Figure S5A), while the inward-facing model had more poses towards the bottom (Supplementary Figure S5B). The outward-facing model also sampled W82-W84 inter-indole angles and distances with larger values than any other model (Supplementary Figure S5). For the AF2 intermediate conformation, urea poses concentrated at the middle of the pathway (Supplementary Figure S5C). This conformation sampled smaller inter-indole angles than the inward-facing model. However, most W82-W84 orientations were separated by 6 Å or less, indicating closer positioning of these residues (Supplementary Figure S5C), thus, the groove between W82 and W84 identified in the putative AF2 binding site was maintained. Interestingly, when comparing the reciprocal organization of the indole groups in W82 and W84 in the three conformations (Figure 3), we noticed only a small overlap in the angles sampled by each conformation. The inter-indole angles only overlapped in the region between 45° and 67.5° (Figure 3A). This suggests that the presence of urea limits the conformational space of W82 and W84, favoring the passage from one conformation to the other.

### 2.2. Site-Directed Mutagenesis of Identified Residues in the Putative Binding Site

The functional importance of residues W82, W84, N279, and T282 is supported by the fact that they are conserved among UreA and its characterized orthologs (Supplementary Figure S6). Moreover, W82 is one of the residues identified in our previous work as putatively involved in substrate binding, recognition and/or translocation. We showed then that the W82A mutation resulted in loss of function, while W82F led only to a partial impairment of UreA transport. Notwithstanding, its affinity for urea remains unchanged; hence, the mutation affects the translocation but not the affinity for urea. Moreover, the affinities for 2-TU, ACM, and guanidine are augmented [16]. This could be explained in terms of the wider space, which would result between W84 and a Phe residue in position 82, instead of a Trp. On the other hand, N279 and T282 lay near positions 82 and 84, are exposed to the solvent, and localize in TMS 7, which is also cognate to a vSGLT segment, which bears elements relevant for substrate binding and translocation (Figure 3 and Supplementary Figure S2).

To test the role of these residues in urea binding and/or translocation, we constructed mutant strains bearing non-conservative (W84A, N279A and T282A) and conservative (W82Y, W84F, W84Y, N279Q and T282S) substitutions. Since all the mutated versions of UreA are fused to GFP, the subcellular localization of the transporter can be monitored by epifluorescence microscopy. In all cases UreA-GFP fusions show a normal localization in the cell membrane. None of the assessed mutations cause differences in UreA levels as shown through Western blots assays (Supplementary Figure S7). Growth tests on urea as sole nitrogen source, as well as resistance assays on 2-TU, and kinetics of urea uptake, alone or in the presence of the urea analogues were assessed. In 2-TU, the carbonyl oxygen is substituted by a sulphur atom, which should reduce the interaction with hydrogen bond donors within the binding site. Moreover, the bigger size of sulphur should also impair stacking interactions. In ACM, a methyl group substitutes for a primary amine, hence reducing the hydrogen donor capacity and favoring hydrophobic interactions within the binding site. Thus, these variations in the functional groups of the ligand can provide additional insights on the interaction modes of urea with its intermediate-state binding site and the effect of the different mutations.

**Figure 4:**
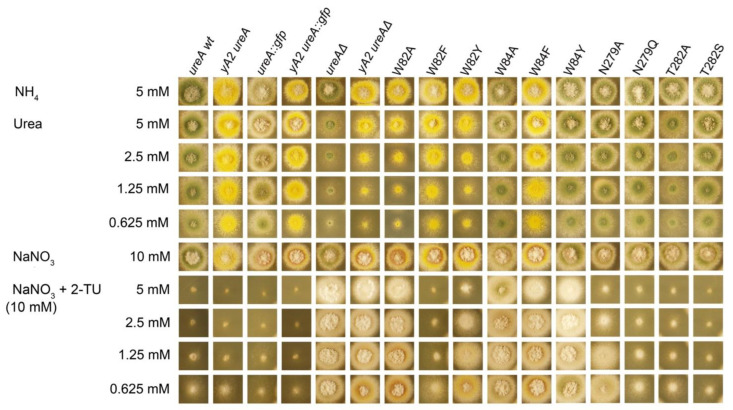
Growth phenotypes of mutant UreA strains. Mutant strains were grown at 37 °C for 48 h on 0.625–5 mM urea as nitrogen source or on 0.625–5 mM 2-thiourea (2-TU) with 10 mM sodium nitrate (NaNO_3_) as nitrogen source to test resistance to the analogue. Growth on acetamide as sole nitrogen source is not shown as no obvious phenotype of growth deficiency is evident when comparing *wt* and *ureA*Δ in all conditions tested. Growth on 5 mM ammonium (NH_4_) and 10 mM NaNO_3_ are used as controls. Wild type (*wt*) and *ureA*Δ strains are shown as positive and negative controls, respectively. Similar results were obtained at 25 °C (Supplementary Figure S8).

Mutant W82Y shows a partial loss of function phenotype, both on urea (0.625–5 mM) and 2-TU (0.625–5 mM) (Figure 4). Regarding W84 mutants, strains carrying mutations W84A and W84F present a slight growth defect on urea. The ^14^C-urea initial apparent uptake rates (V_o_) in those mutants are 15–25% with respect to the wild-type (*wt*), while urea binding affinities, reflected in calculated *K_m_*s, fall in a range between 1.6 and 7.4 times lower than in the *wt* (Table 1). Thus, these results suggest that the mutations would have an effect not only at the level of affinity but also at the level of translocation.

The W84Y mutation renders an almost *wt* phenotype on urea, with a *K_m_* 6 times higher than that of the *wt*. Notwithstanding, the initial urea uptake rate is approximately half of that observed in the *wt*. These results suggest that changing a W into an Y may compensate for the loss in affinity for urea with a more efficient translocation.

On 2-TU all the mutants show a resistant phenotype, which is indistinguishable (W84A) or slightly less (W82Y, W84F and W84Y) than that of the *ureAΔ* strain. Accordingly, in all cases affinity diminishes to less than half that of the *wt*, except for the 1.5-fold drop observed for the N279Q mutant. Similar results were obtained for ACM affinities, yielding affinities that are at least half of that measured for the *wt* strain, except for W82Y, which presents an affinity increased to just over the double (Figure 4; Table 1). Thus, taken together, these results highlight the involvement of residues W82 and W84 in substrate recognition, selectivity, and translocation. Concerning mutations on N279, the conservative change into a Q causes a very mild loss-of-function phenotype of the mutant strain both on urea and 2-TU. The determined urea transport V_o_ was ≈70% and affinities for urea and 2-TU were, respectively, 3.5- and 1.5-fold lower than that of the *wt.* The growth phenotype associated with the non-conservative mutation N279A is also barely impaired with respect to that of the *wt* on urea but a little bit more pronounced in 2-TU. Accordingly, the urea V_o_ and *K_m_* values do not differ significantly from those of the *wt*, while the calculated *K_i_* for 2-TU is almost twice as that of the *wt*. The affinity for acetamide, on the contrary, improves in both mutants, with a *K_i_* ≈ 2.7-fold lower for the N279Q mutant and ≈4.6-fold lower for the N279A mutant, as compared to the *wt*. These results suggest that N279 is part of the UreA binding site, with a role in substrate selectivity.

Finally, the conservative change T282S does not produce distinctive phenotypes on urea and 2-TU, and ^14^C-urea presents an initial uptake rate of ≈65% with respect to the wild type. Notwithstanding, the mutation causes a 5-fold drop in affinity for urea and >2-fold drop in that for 2-TU. The affinity for ACM remains almost unchanged. Strains bearing the non-conservative T282A mutation show a significant growth defect on urea, with a V_o_ that is only 14% and a *K_m_* 3-fold lower than those of the *wt*, while resistance on 2-TU is slightly better. Determined affinities for 2-TU and acetamide showed a 2 and 1.6-fold decrease, respectively. Therefore, T282 would also participate in substrate binding, translocation, and selectivity in UreA.

### 2.3. Insights into Ligand Binding by the Intermediate State of UreA

The outcomes of non-conservative and conservative mutations of W82, W84, N279, and T282 suggest they have important roles at the level of ligand binding and urea transport. To understand how these residues interact with ligands, we docked them using Autodock Vina into the predicted intermediate state of UreA. As observed before, this structure’s shape concentrates docking poses at the putative binding site, better than the other modeled conformations (Supplementary Figure S5). Thus, we centered docking calculations on this area and kept the four residues (W82, W84, N279, and T282) and their mutations (W82Y, W84A, W84F, W84Y, N279A, N279Q, T282A and T282S) flexible. For the sake of completeness, we added the W82F substitution, previously characterized in Sanguinetti et al. (2014) [8]. This mutation only slightly interfered with urea translocation, while it improved the *K_i_* values for 2-TU and ACM. Thus, inserting W82F helps to populate our docking calculations with a challenging case.

**Figure 5:**
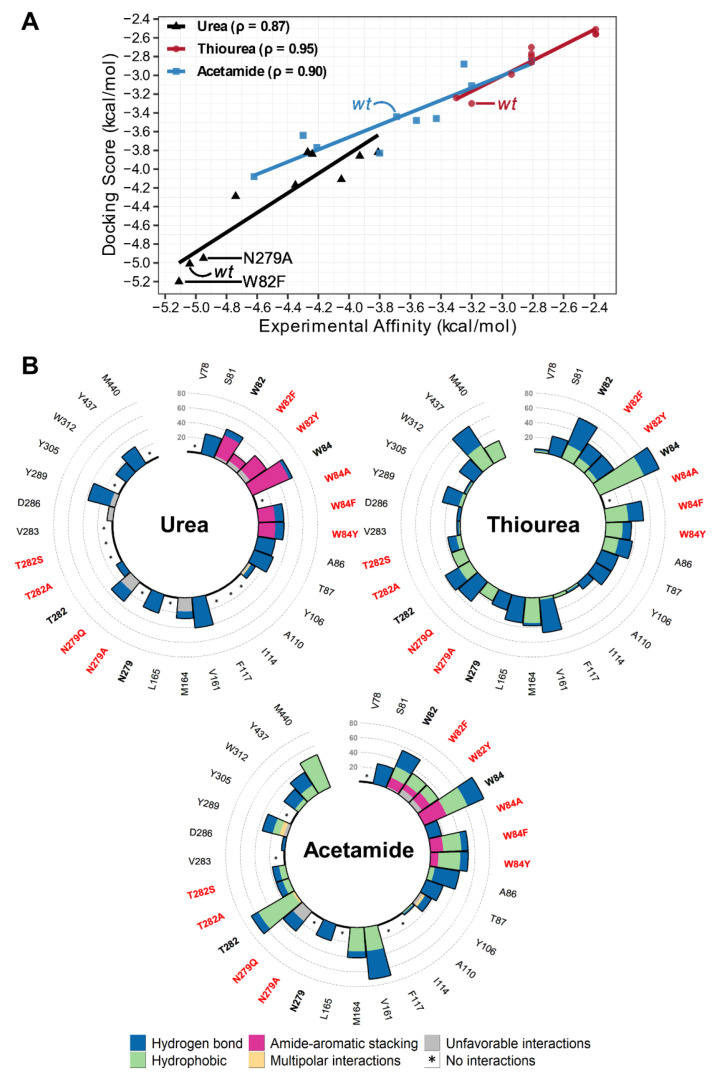
Docked urea, 2-TU, and ACM correlation and interaction profiles. (**A**) *wt* and nine mutations’ docking scores and experimental affinities correlated well for all ligands. Spearman correlation was 0.87, 0.95, and 0.90 for urea, 2-TU, and ACM, respectively. We highlighted all *wt* affinities, and urea results for W82F and N279A that have close affinity to *wt*. (**B**) Urea, 2-TU, and ACM had varied interaction profiles. Urea’s most prevalent interactions were amide-aromatic stacking and hydrogen bonding. Most residues in 2-TU and ACM have more hydrophobic interactions, while ACM’s W82 and W84 residues also achieved amide-aromatic stacking similar to urea.

To validate the intermediate state of UreA and our docking results, we correlated docking scoring affinities with the experimental affinity of urea, 2-TU, and ACM. Autodock Vina’s scoring function overestimates binding affinities despite calculating binding poses well. Thus, docking scoring affinities were predicted with Vinardo, which improves ranking and score accuracy when combined with the Vina algorithm. Average docking scores, based on three top scoring poses from the *wt* that were similarly predicted for the mutations (Supplementary Figure S9), showed a remarkable correlation with experimental binding data for all systems, with urea reaching a correlation (ρ) of 0.87, while 2-TU and ACM reached ρ = 0.95 and ρ = 0.90, respectively (Figure 5A). The agreement between theoretical and experimental results determined for 9 mutants and three different ligands provide further support to our initial hypothesis about the existence of an intermediate state binding site in UreA.

To provide a more comprehensive view on the molecular recognition between UreA and the ligands, we analyzed the common protein–ligand interactions for the chosen docking poses in all evaluated mutant and *wt* systems, using the program LUNA [40]. Overall, urea interacted with the binding site residues mostly by hydrogen bonds and amide-aromatic stacking, while 2-TU and ACM did so mainly by establishing hydrophobic contacts (Figure 5B). Residues W82, W84 and their substituting residues were consistently found among top interacting residues in all systems, except for W84A (for all ligands) and W82F in the case of urea. As with urea, W82 and W84 showed some amide-aromatic stacking interactions with ACM but with less frequency, while no such interactions occur with 2-TU. Residue N279 was not a top interacting residue, but it formed hydrogen bond interactions with all ligands, and its mutations (N279A and N279Q) abolished or reduced the interaction frequencies. Residue T282 interacted more with ACM, while its mutations (T282A and T282S) eliminated contact with urea and reduced interactions with both 2-TU and ACM. Other highly interacting residues were A86, V161, Y289, and Y437. For the latter two, urea showed more contact with Y289, while 2-TU and ACM further interacted with Y437.

These differences in the interaction profiles suggested that affinity variations may reflect how the binding site interacts and recognizes the ligands. When comparing ligand interaction frequency profiles of each mutation with that of the *wt*, some substitutions shifted the interaction frequencies for some of the analyzed residues. For urea (Supplementary Figure S10), the mutations that maintained *wt* affinity (W82F and N279A) featured W84 as the top frequency residue, interacting largely by amide-aromatic stacking, and conserved interactions were displayed with Y289 and Y437 as well. For ACM (Supplementary Figure S11), mutations that increase affinity (W82F/Y and N279A/Q) present amide-aromatic stacking interactions with both W82, W84 or only one of them, and extensive hydrophobic contacts with T282. For 2-TU, all mutations led to lower affinities, which correlates with the lack of amide-aromatic stackings interactions and shifts in hydrogen bonds and hydrophobic interactions with residues W82, T282, Y289 and Y437 (Supplementary Figure S12).

## 3. Discussion

In Sanguinetti et al. (2014) [8], the construction of inward- and outward-facing cavities using comparative modeling, together with site-directed and conventional random mutagenesis, revealed a significant role of conserved residues W82, Y106, A110, N275, D286, and Y437. These residues are in TMSs 2, 3, 7 and 11 that correspond to putative substrate binding and recognition regions in other characterized SSS transporters. Transporters such as UreA are supposed to operate on the basis of a “rocking bundle” mechanism, which alternates between inward- and outward-facing conformations, as a result of substrate binding. Therefore, there must be intermediate conformations of UreA accompanied by selectivity filter mechanisms for substrate translocation.

Usually, comparative modeling and other prediction approaches are based on existing structure templates in specific states. Thus, multiple states on systems that “flip” between conformations to perform their functions are precluded. Many efforts have been made to predict multistate structures that are not influenced by one specific state [33,34,35,36,41,42]. Del Alamo et al. (2022) [21] attempted to expand the use of AI-based AF2 beyond the structural prediction of an already determined biological state for a structure, especially for membrane transporters and receptors, since some previous AF2 predictions were observed to be in intermediate state conformations [33,34]. With the use of this approach, AF2 was effectively driven to sample alternative conformations of transporters and G-protein-coupled receptors that were not present in the AF2 training set. In this study, we used AF2 to build an alternative conformation of UreA and further characterize this family of proteins. Since no experimental evidence of UreA’s transitions is available and structural information of other homologues are scarce, we would like to emphasize that the theoretical conformations discussed here are not to be considered as high-resolution structures. However, good correlation found between theoretical and experimental data in this and our previous publication [16] suggest the models are reliable up to the single amino acid level.

Our UreA structure prediction with AF2 achieved models that showed conformations lacking inward- and outward-facing cavities and large flexibility on residues W82, W84, N279, and T282. Outward- and inwards-facing models revealed considerable distancing of the TMSs 2, 3, 7 and 11, while in the AF2 predicted structure TMSs 2 and 7 showed narrower distancing. Thus, the AF2 prediction suggested a model in an intermediate conformation between the outward and inward-facing ones. This proposed intermediate conformation, with a narrower pathway, showed a putative binding site composed of residues W82, W84, N279, and T282, that could bind the substrate and stabilize the protein structure. These residues are conserved among UreA and its characterized orthologs in fungi and plants, which supports their functional relevance. This hypothesis was further explored with the help of molecular docking with flexible side chains of W82, W84, N279, and T282. As a result, for all the models W82 and W84 movements were shown to sample angles between 45° and 67.5°, mostly staying close to each other (≤6 Å). This overlap in the sampling frequency of the angles showed that the models have similar space to move these residues and the presence of urea could influence a gating mechanism ruled by the ability of W82 and W84 to face each other. The experimentally assessed effect at the level of substrate binding, recognition, and translocation of non-conservative and conservative substitutions of residues W82 and W84 in TMS 2, and N279 and T282 in the opposite TMS 7, in UreA, support the conclusion that they belong to the substrate binding site.

In our previous work, W82 was suggested to have a role in substrate translocation, which is supported in this work by the outcome of the W82Y mutant, since while the drop in urea affinity is ≈50%, the mutant strain retains only ≈25% of its transport capacity. Additionally, apparent affinities for 2-TU (diminished to less than half) and ACM (augmented to more than twice) would confirm an involvement at the level of substrate selectivity. In the case of the W84 residue, a drop in affinity was the general tendency for urea and its two analogues. Thus, a major role at the level of substrate binding could be attributed to this residue. Regarding residue N279, the conservative substitution by glutamine led to diminished affinities for urea and 2-TU, but an increased affinity for ACM, while the non-conservative N279A change caused a drop in affinity for 2-TU and a rise in this parameter for ACM, and no significant change was observed in the case of urea. In sum, these results denote the involvement of this residue in both substrate binding and selectivity. Finally, both conservative and non-conservative mutations of residue T282 provoked affinity drops for the three tested UreA ligands. Accordingly, urea transport rate diminished in both mutants. It can then be concluded that T282 is involved in substrate binding. In all cases growth tests accompanied the changes in kinetic parameters. It is worth noting that the effect on 2-TU resistance is clear only when *K_i_* values are >2000 µM.

As the use of AF2 models for docking-based purposes is still on debate [43,44,45], we validated our intermediate-like model using site-directed mutagenesis outcomes for urea, 2-TU, and ACM. The average score obtained was not based on a specific binding mode, but on three poses found in comparable positions in the putative binding region for *wt* and the nine mutations, providing a more dynamic study of the protein–ligand interactions (Supplementary material, Figure S8). The chosen poses were accompanied by movements of the W82, W84, N279, and T282 side chains, providing the prospective impact of urea, 2-TU, and ACM at the binding site. Our approach correlated well with average experimental binding data for all systems (ρ ≈ 0.90). Although the combination of the intermediate model and docking can help us to understand how UreA residues possibly affect ligand selectivity, we are limited by the simplistic manner that these approaches deal with solvent and proton effects in ligand binding [46,47]. Thus, bridging, hydration, displacement of water molecules, and the presence of explicit H^+^ protons are aspects in UreA function not investigated by the computational models applied here, but known to be relevant in urea related complexes [48].

Since urea does not create hydrophobic contacts, such as 2-TU and ACM, according to non-bonded interaction profiles, the lack of this specific interaction may explain the exquisite UreA’s affinity and selectivity for urea. In addition, formation of amide-aromatic stacking with W82 and W84, predominantly with urea and in some cases ACM, is in agreement with the observed loss of binding affinity determined for these ligands after the site-directed mutagenesis of these residues. We hypothesize that ligand stacking can alter W84 orientation, allowing urea to enter into the groove that forms the putative binding site. W84 orientation is stabilized by Y437 (previously proposed to have a role in substrate selectivity [16]) to keep one end of the “gate” closed. Once urea is “sandwiched” by W84 and W82 and stabilized with the help of other residues, such as T87, Y106, V161, N279, Y289, and Y437, it can establish the same kind of interaction with W82. This interaction with W82 pushes the residue to change orientation, this W82 orientation might be kept by interactions with N279 and Y289, thus opening the pathway for urea translocation. In this scenario, T282 may briefly interact with W84 to keep the “gate” open and with W82 to stabilize its “sandwiched” orientation, instead of forming interactions with urea. Thus, we speculate that T282 “attracts” ACM, interfering with its amide stacking interactions with W82 and locking the “sandwiched” orientation inhibiting translocation. Since 2-TU docking experiments did not display amide-aromatic stacking interactions, we suggest that 2-TU presence may hinder the hypothesized gate mechanism. Therefore, our hypothesis suggests a highly specific ligand-gated mechanism in UreA that is not effectively obeyed by 2-TU and ACM.

## 4. Materials and Methods

### 4.1. Structure Prediction

AF2 models were generated using the slightly simplified and automated version from AlphaFold Colab [32,49]. This Google Colaboratory webserver is a Jupyter Notebook that runs on local machines through the internet. We used the AF2 Colab with default settings that includes no templates and a smaller multiple sequence alignment (MSA) database (UniRef100 and an environmental sequence collection) with the MMseqs2-based search. Molecular models of closed UreA inward- and outward-facing conformations were built using the structures of vSGLT of *V. parahaemolyticus* [22] and Mhp1 from *M. liquefaciens* [23], respectively, as templates with MODELLER 9.12 [50]. These models were previously generated and discussed in Sanguinetti et al., 2014 (see [16] for more details).

### 4.2. Molecular Docking

Autodock Vina version 1.2.3 [38,39] was used as the docking program with the Vinardo scoring function [51]. This latest version of Autodock Vina was chosen due to its improved algorithm and the ability of sampling flexibility of protein side chains without increasing computer processing time, while Vinardo provides ranking and score accuracy. All UreA conformations and ligands were prepared using the MGLTools scripts [52] and analyzed with the ViewDock tool from the UCSF Chimera [37] version 1.16. In all docking runs, residues W82, W84, N279, T282, and their mutations had flexible side chains, while all the remaining amino acids remained spatially constrained. For the urea placement runs, a grid box of 18.0 × 30.0 × 20.0 Å was centered around residues W82 and W84. Five random seeded runs sampled urea places. To get different results, the energy range was increased to 50 kcal/mol and the maximum number of modes set to 100, while the exhaustiveness parameter was kept at 10. Positive scores were removed from the analysis. For the docking urea, 2-TU, and ACM in the AF2 conformation, a smaller grid box of 15.0 × 15.0 × 15.0 Å was used. Three runs with default options, except exhaustiveness that was set to 10, were utilized to collect data.

### 4.3. Structural Analysis

We used the Morph Conformations capability from UCSF Chimera [37] to build UreA theoretical trajectories between two conformations. These theoretical trajectories were constructed utilizing linear and ramp-down interpolation rates and types, respectively. A total of 60 intermediate morphing proposed conformations were produced. For the urea placement analysis, angles, and distances between W82 and W84 were determined using the VMD [53] script *pistack.tcl* from *gmxtools* (10.5281/zenodo.6408973). The script determines the rings’ planes, computing their angles from their normal vectors and distances from the rings’ center. We added in the script the calculation of urea’s center of mass to obtain its channel position. Final docking scores for urea, 2-TU, and ACM scores were determined using the top three poses of each run for *wt* systems. For the mutations, the average docking score was composed of the same or similar poses as the *wt* systems. The average ligand docking score was compared to the average experimental data using the spearman correlation ranking approach. These final protein–ligand complexes were submitted to LUNA [40] for non-covalent interaction prediction. This tool helped us to determine the frequency of interactions between the three ligands and UreA residues. All plots were created with the R program [54].

### 4.4. A. nidulans Strains, Media and Transformation Procedures

Strains were grown on *A. nidulans* standard complete and minimal media (MM) [55,56], with addition of supplements at standard concentrations, when necessary (https://www.fgsc.net/; accessed on 9 November 2022). *A. nidulans* strains used and generated in this study are listed in Table S1. Gene symbols are defined in https://www.fgsc.net/Aspergillus/gene_list/loci.html (accessed on 9 November 2022). Urea (0.6–5 mM), NaNO_3_ (10 mM), ammonium L(+)-tartrate (5–10 mM) or proline (5 mM) were used as N-sources; 2-thiourea was used in concentrations of 0.6–5 mM. *A. nidulans* strains were transformed as described in [57].

### 4.5. Construction of ureA Mutants by Site-Directed Mutagenesis

Site-directed mutagenesis was carried out by Fusion-PCR technique [57] on DNA from a strain carrying a *ureA::gfp* fusion (MVD 10A), using KAPA HiFi DNA polymerase (Roche Molecular Systems, Pleasanton, CA, USA) and the primers listed in Table S2. Resulting 7 kb fusions were amplified with nested primers Ure5-N and Ure3-N. PCR products were purified with Monarch Nucleic Acid Purification Kits (New England Biolabs, Ipswich, MA, USA). Resulting construct was introduced in the MVD13A or MVD14A strains (*ureA∆::riboB pyrG89 pyroA4 riboB2 nkuA∆::argB veA1*, the latter also bearing mutation *yA2*) and transformants were selected on sucrose MM selective plates, containing riboflavin and pyridoxine, but not uridine and uracil, at 37 °C. Purified transformants were tested for their ability to grow on different concentrations of both urea and 2-TU. The unique integration to the *ureA* locus was assessed by Southern blots. The resulting mutations were checked by sequencing (Macrogen Inc., Seoul, Republic of Korea).

### 4.6. [^14^C]-Urea Uptake Measurements

Radiolabelled [^14^C]-urea (55.0 mCi mmol^−1^) was purchased from Moravek Biochemicals, Brea, CA, USA. [^14^C]-urea uptake assays were performed in *A. nidulans* germinating conidiospores. Initial velocities were measured after 2 min of incubation at 37 °C with a [^14^C]-urea concentration of 1.0 μΜ, and are expressed as % of the *wt* value, in pmoles min^−1^/10^7^ conidiospores. Apparent *K_m_*/i values were obtained directly by performing and analyzing [^14^C]-urea uptakes (Prism 3.02: Graph Pad Software, Inc., San Diego, CA, USA), from IC_50_ values in the presence of various concentrations (0.5–3000 μM) of non-labelled substrates, as previously described [4,16,58]. In all cases, the Hill coefficient was close to −1, consistent with competition for a single binding site. The *K*_i_ values are then calculated from IC_50_ values, based on the equation: *K*_i_ = IC_50_/[1 + (S/*K*_m_)] [59] and in this context it should also be taken into account that a *K*_i_ only implies binding but does not indicate actual transport across the membrane. Transport assays were carried out in at least three independent experiments, in triplicates for each concentration or time point. Background uptake values were corrected by subtracting values measured in the deleted mutants (UreAΔ). Standard deviation was <20%.

### 4.7. Protein Extraction and Western Blot Assays

Total protein extracts were obtained from 200 mg of grinded mycelia, as described in Apostolaki et al., 2012 [60] and protein concentration determined by using the Pierce BCA Protein Assay kit (Thermo, Waltham, MA, USA). 50 µg of total proteins were separated by SDS-PAGE (10% (*w*/*v*) polyacrylamide gel) and electroblotted onto a nitrocellulose membrane (0.45 Micron, Thermo Scientific, Waltham, MA, USA), which was then blocked with 5% non-fat dry milk. Immunodetection was performed with primary mouse monoclonal antibodies against anti-GFP (Roche Molecular Systems) or against anti-tubulin (clone AA4.3, Developmental Studies Hybridoma Bank, DSHB, Iowa, IA, USA) and a secondary sheep anti-mouse IgG HRP-linked antibody (GE Healthcare, Chicago, IL, USA). Blots were developed with the Amersham ECL Western Blotting detection kit and images were acquired with the GBox Chemi XT4 System and analyzed with the GeneSys software (Syngene, Bengaluru, India).

### 4.8. Epifluorescence Microscopy

Samples for epifluorescence microscopy were incubated in glass-bottom dishes in liquid minimal media with proline (5 mM) as nitrogen source and appropriate supplements at 25 °C for 14–16 h and protected from light. Observations were done in an Olympus inverted microscope CKX31, which belongs to the Advanced Bioimaging Unit, Institut Pasteur de Montevideo, and is equipped with a U-MNIBA3 filter for GFP and a Hamamatsu Orca Er camera, and the Micro-Manager software [61] (https://micro-manager.org/, Madison, WI, USA).

## Figures and Tables

**Table 1:** Summary of the assessed UreA mutations studied and determined kinetic and affinity data. V (%): initial uptake rates expressed as % of those in the *wt*, in pmoles min^−1^/10^7^ conidiospores.

Allele	Location in Protein	V_o_ (%)	Urea *K_m_* (µM)	Thiourea *K_i_* (µM)	Acetamide *K_i_* (µM)
*wt*		100	26 ± 2	528 ± 42	237 ± 14
W82F *	TMS2	50 ± 8	23 ± 5	447 ± 24	198 ± 12
W82Y	TMS 2	25 ± 2	42 ± 5	>1000	101 ± 16
W84A	TMS 2	20 ± 2	193 ± 12	>2000	489 ± 39
W84F	TMS 2	15 ± 2	96 ±13	>2000	524 ± 42
W84Y	TMS 2	46 ± 4	159 ± 11	>2000	>1000
N279A	TMS 7	100 ± 5	30 ± 3	>1000	51 ± 5
N279Q	TMS 7	70 ± 3	91 ± 6	813 ± 59	87 ± 9
T282A	TMS 7	14 ± 2	80 ± 27	>1000	361 ± 85
T282S	TMS 7	65 ± 3	130 ± 22	>1000	290 ± 12

* Data reported in Sanguinetti et al. (2014) [8].

## Data Availability

Not applicable.

## Supplementary Materials

The following supporting information can be downloaded at: https://www.mdpi.com/article/10.3390/ijms232416039/s1.
