# Supplementary material for: Substrate Recognition Properties from an Intermediate Structural State of the UreA Transporter
Source: Int J Mol Sci. 2022 Dec 16;23(24):16039. doi: 10.3390/ijms232416039 (PMC9783183; doi:10.3390/ijms232416039)
Supplement: Supplementary file 1 [file ijms-23-16039-s001.zip › Supplementary material.pdf]

Supplementary material

Substrate recognition properties from an intermediate structural state of the UreA transporter

Manuel Sanguinetti [1,†], Lucianna Helene Silva Santos [2,†], Juliette Dourron [1], Catalina Alamón [1,4], Juan Idiarte [1,5], Sotiris Amillis [3], Sergio Pantano [2,*], Ana Ramón [1,*]

1 Sección Bioquímica, Departamento de Biología Celular y Molecular, Facultad de Ciencias, Universidad de la República, Iguá 4225, Montevideo 11400, Uruguay
2 Biomolecular Simulations Group, Institut Pasteur de Montevideo, Mataojo 2020, Montevideo 11400, Uruguay
3 Neurodegeneration Laboratory, Institut Pasteur de Montevideo, Mataojo 2020, Montevideo 11400, Uruguay
4 Columbia University Irving Medical Center, Columbia University, New York, NY 10032, USA
5 Department of Biology, National and Kapodistrian University of Athens, Panepistimioupolis, 15784 Athens, Greece
* Correspondence: spantano@pasteur.edu.uy (S.P.); anaramon@fcien.edu.uy (A.R.); Tel.: +598-2522-0910 (S.P.); +598-25252095 (A.R.)
† These authors contributed equally to this work.

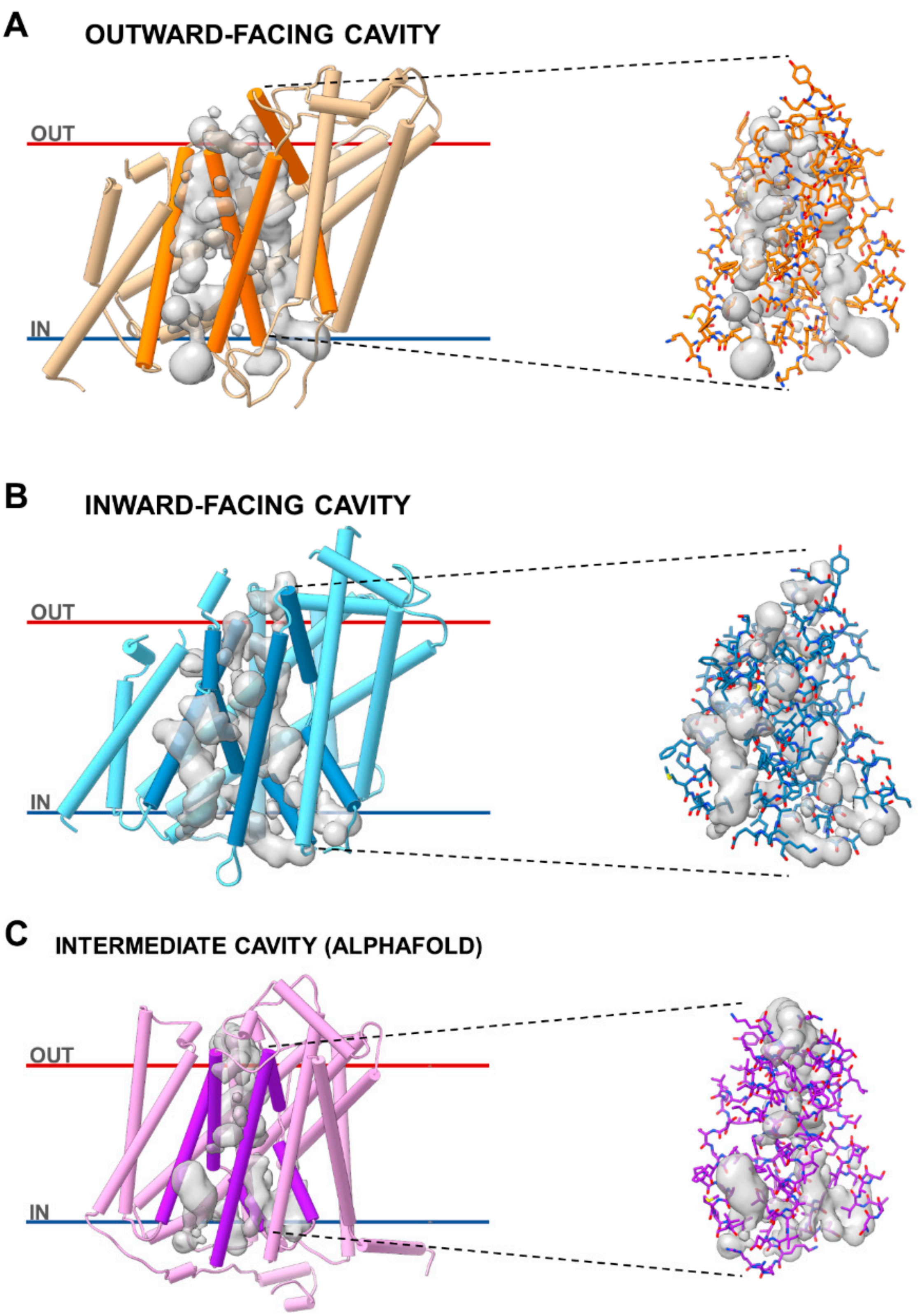

Figure S1. Volume of the possible urea pathway formed by the TMSs 2, 3, 7 and 11. Space between TMSs for the outward-facing cavity model (A) displayed a slighter higher volume at the top of the structure, while for the inward-facing cavity model (B), volume was more condensed at the bottom of the structure. In the AF2 predicted model that resembles an intermediate conformation (C), the pathway was narrower with less voluminous vestibular space between the TMSs. Molecular cavities and indentations were calculated with the Surfnet tool from UCSF Chimera.

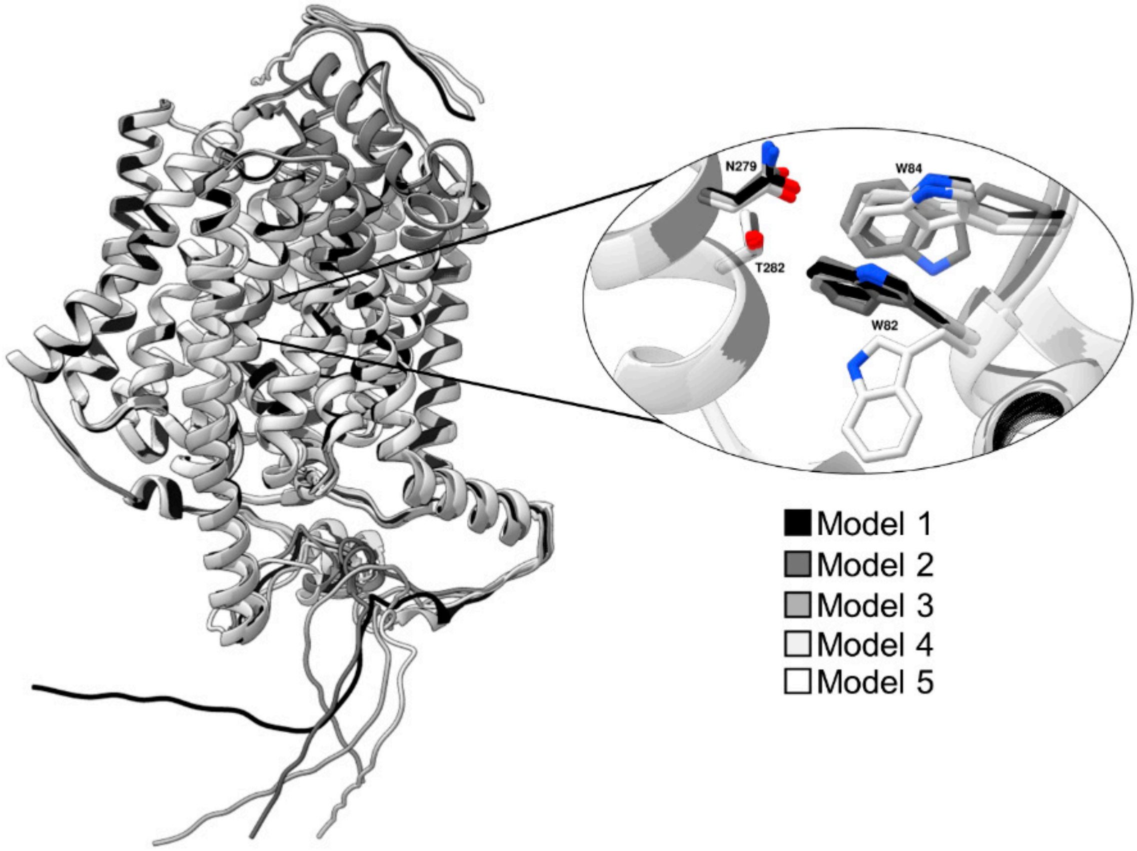

Figure S2. Top 5 UreA models created by AlphaFold2. Residues W82 and W84 from TMS 2, and N279 and T282 from TMS 7 are highlighted due to their orientation differences among the models.

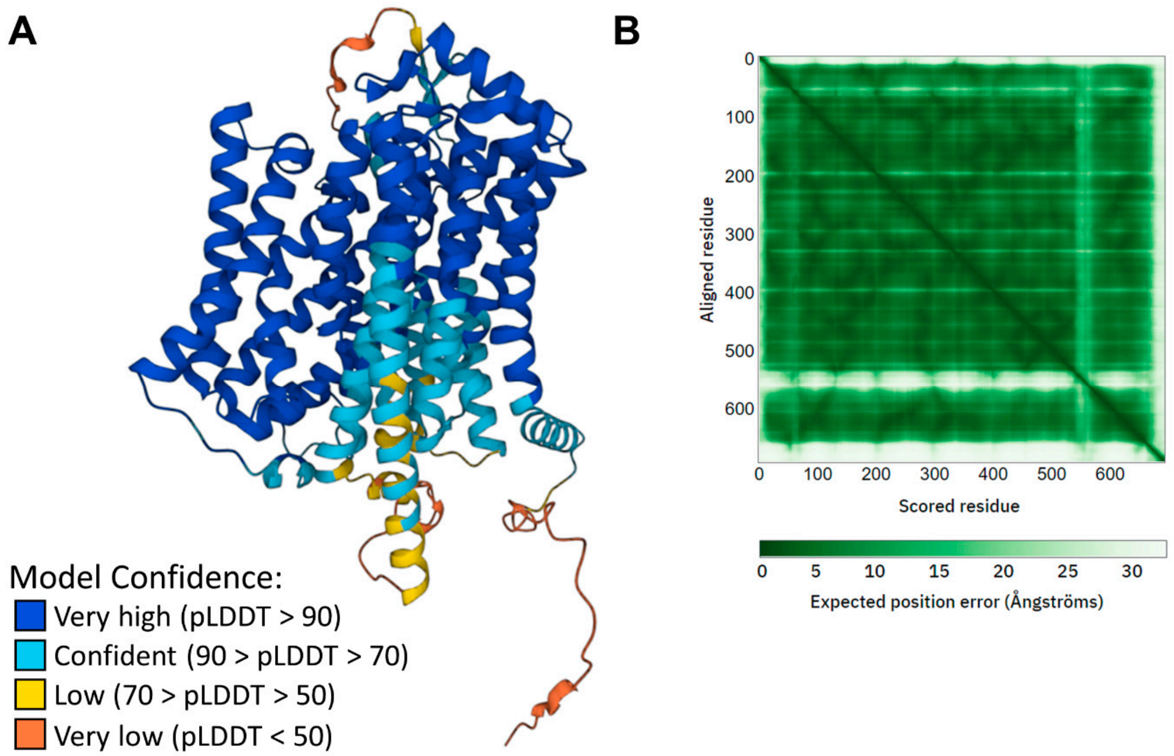

Figure S3. UreA's AlphaFold2 confidence level. A) AlphaFold2 generates a per-residue confidence score (pLDDT) between 0 and 100, ranging from blue (high confidence) to orange (low confidence), as a means of validating the predicted structure. Thus, the overall prediction would fall in a moderate-to-high confidence with a few helix and loops regions in the low confidence level. However, regions below 50 pLDDT might be unstructured in isolation. B) AlphaFold2 also generates a predicted aligned error. This green colored plot indicates AlphaFold2's expected position error at residue X, when the predicted and true structures are aligned on residue Y, dark green (low expected position error) to light green (high expected position error) make up the plot colors. For UreA's model, regions between residues 1-11, 549-565, and 665-663 have very high error values.

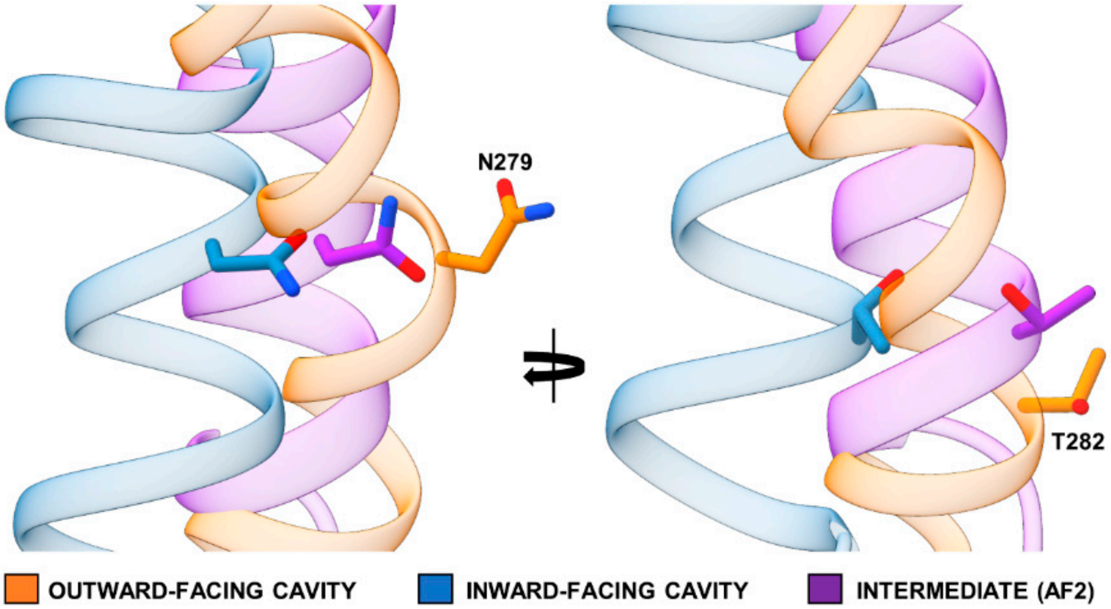

Figure S4. TMSs 7 in the different UreA conformations. From the different conformations, we could observe a separation between TMS 7 and TMS 2, with the AF2 model in an intermediate position. A mobility in the orientation of N279 and T282 could also be observed.

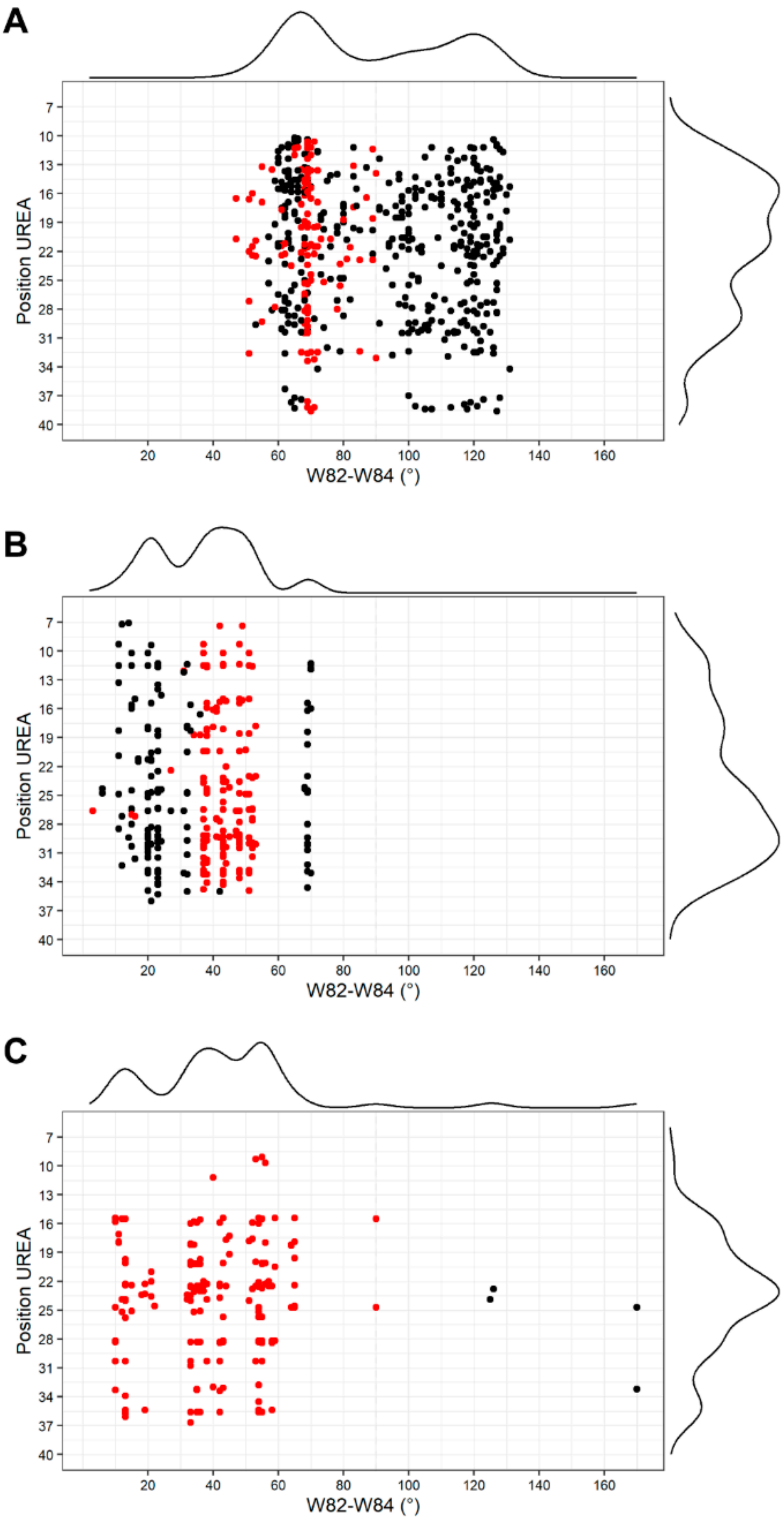

Figure S5. Sampled urea positioning in the UreA pathway in relation to the angles between planes defined by the indole moieties of W82 and W84. All three models sampled urea position throughout the pathway. However, urea poses concentrated at different points in the pathway for each model. For the outward conformation (A), urea concentrated at the top of path. The red points show conformers compatible with aromatic stacking, as they

comprise W82-W84 angles ranging from 0° to 90° and inter-indole distances below 6 Å. For the inward conformation (B), urea poses concentrated at the bottom of the pathway, while for the intermediate AF2 conformation (C), urea concentrated near the middle of the pathway. The outward model had highest angles among the models, while inward and intermediate models sampled similar values. Most of the AF2 poses are compatible W82-W84 with aromatic stacking.

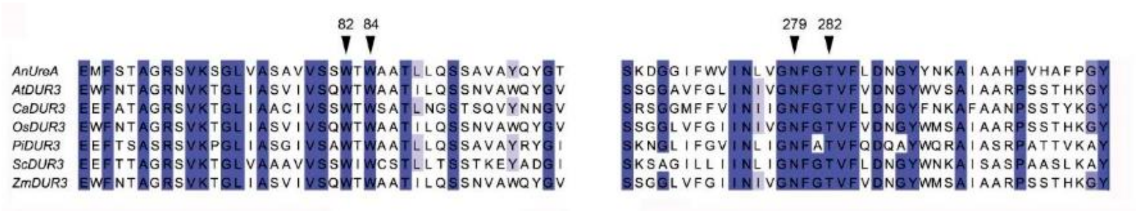

Figure S6. Multiple sequence alignment of UreA and characterized orthologues. Aligned sequences include *A. nidulans* UreA (*AnUreA*, GI: 67516273) and characterized orthologues in fungi and plants— *AtDur3* of *A. thaliana* (GI: 9758728), *CaDur3* of *C. albicans* (GI: 68484979), *OsDur3* of *O. sativa* (GI: 115483686), *PiDur3* of *P. involutus* (sequence kindly provided by Morel et al. 2008), *ScDur3* of *S. cerevisiae* (GI: 51013791) and *ZmDur3* of *Z. mays* (GI: 103629941). For space reasons, only those segments bearing the mutated amino acids in this work (W82, W84, N279 and T282, denoted by black arrows) are shown. Fully conserved amino acids are shaded in blue, and structurally conserved amino acids are shaded in grey.

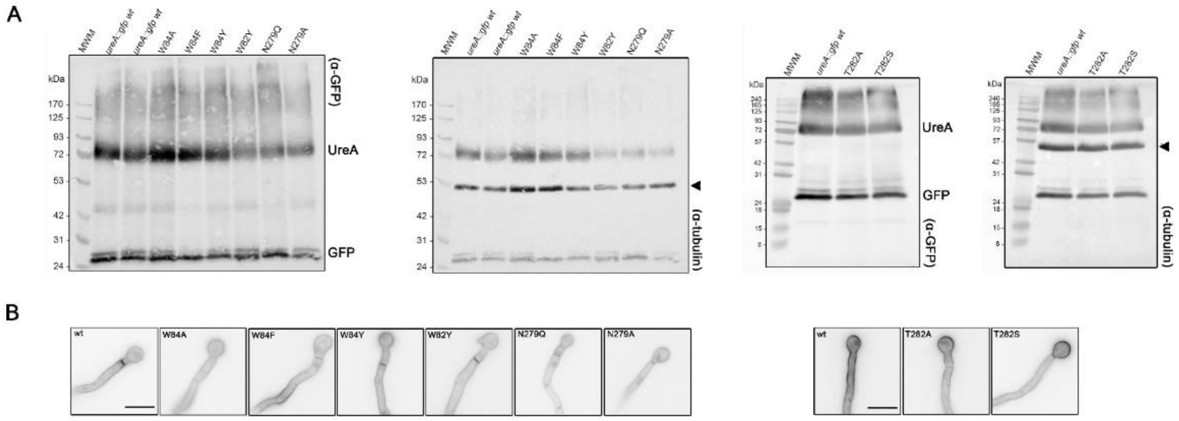

Figure S7. UreA subcellular localization and synthesis is not affected by introduced mutations. A) Western blot analysis of total protein extracts of wild type (*ureA::gfp wt*) and UreA–GFP mutants probed with anti-GFP antibody. Cultures were grown for 14–16 h at 37°C in derepressed conditions (proline as sole nitrogen source). The low mobility band corresponds to intact UreA–GFP and the high mobility band to free GFP (the latter resulting from UreA–GFP turnover). Antibody against tubulin was used as an internal control of loading. B) Epifluorescence microscopy of UreA mutants grown in derepressing conditions. Wild type UreA-GFP (*ureA::gfp wt)* is shown as control. Scale bar, 10 μm.

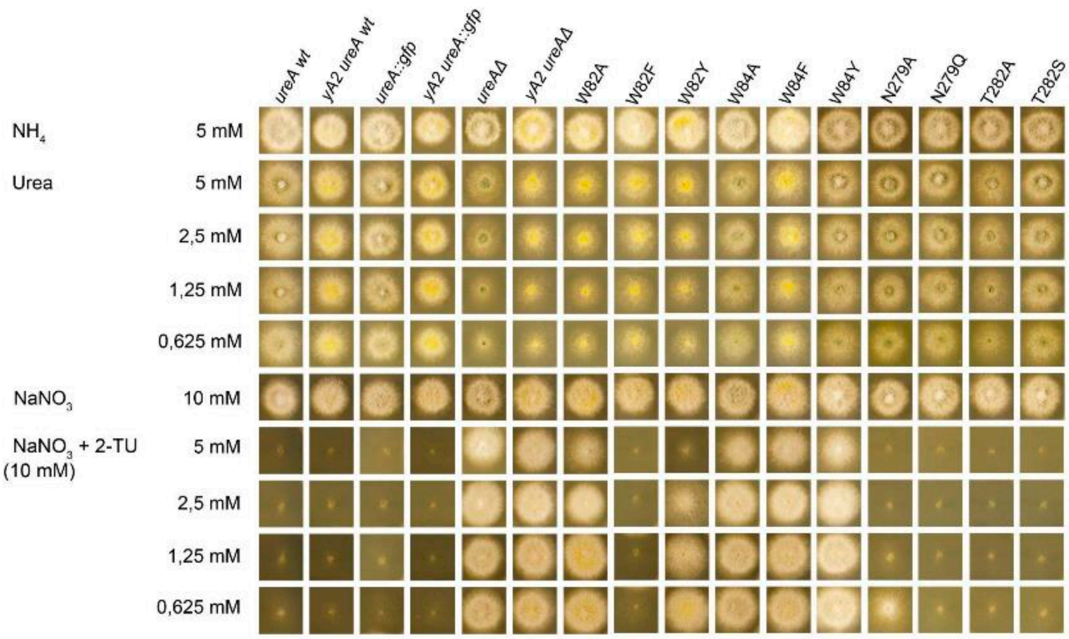

Figure S8. Growth phenotypes of mutant UreA strains at 25 °C. Strains were grown for 72 hours on urea as nitrogen source or on 2-thiourea (2-TU) with 10 mM sodium nitrate ($NaNO_3$) as nitrogen source to test resistance to the analogue. Growth on 5mM ammonium (NH_4) and 10 mM $NaNO_3$ are used as controls. Wild type (*wt*) and *ureAΔ* strains are shown as positive and negative controls, respectively.

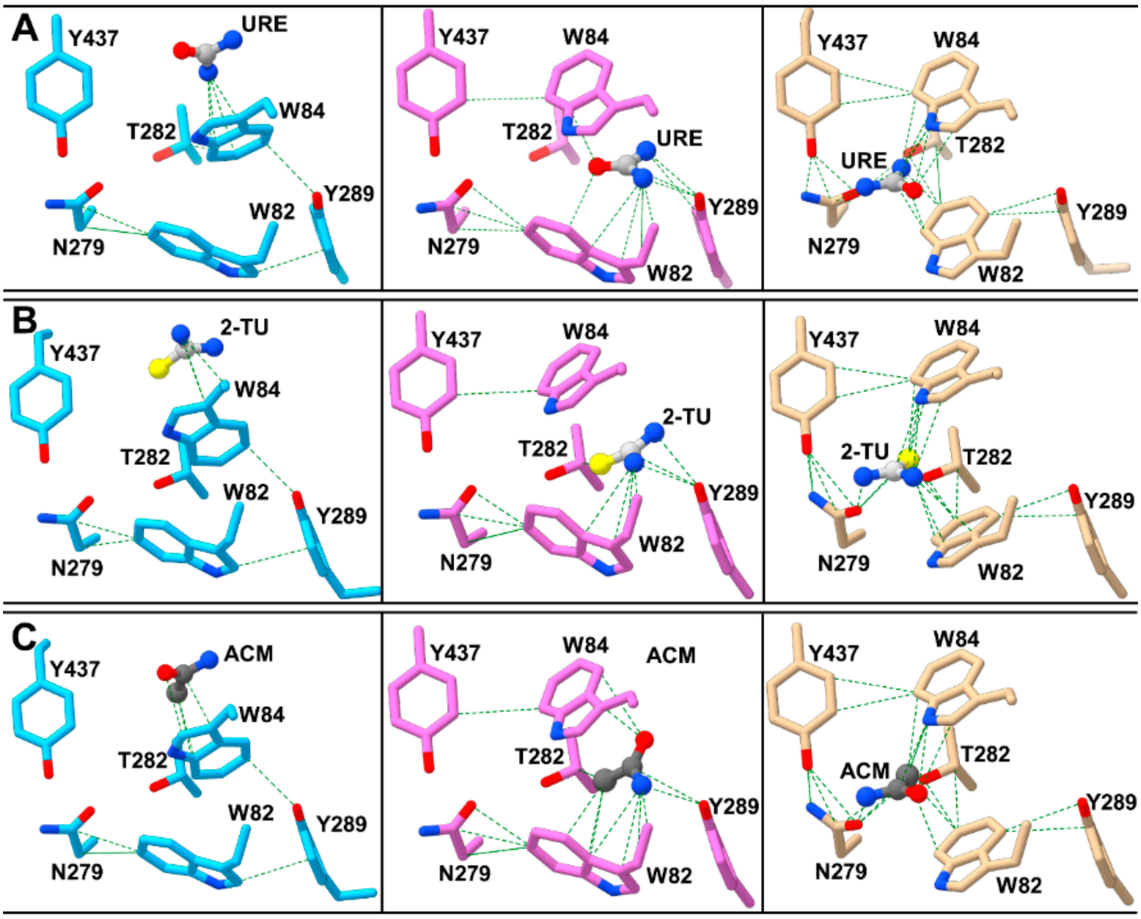

Figure S9. Top scoring docking poses from WT chosen to represent the ligand pathway. (A) Urea. (B) 2-TU. (C) ACM. As all ligands had three almost identical poses, mutant systems were chosen based on these poses. Green dotted lines represent contacts between the residues or ligands.

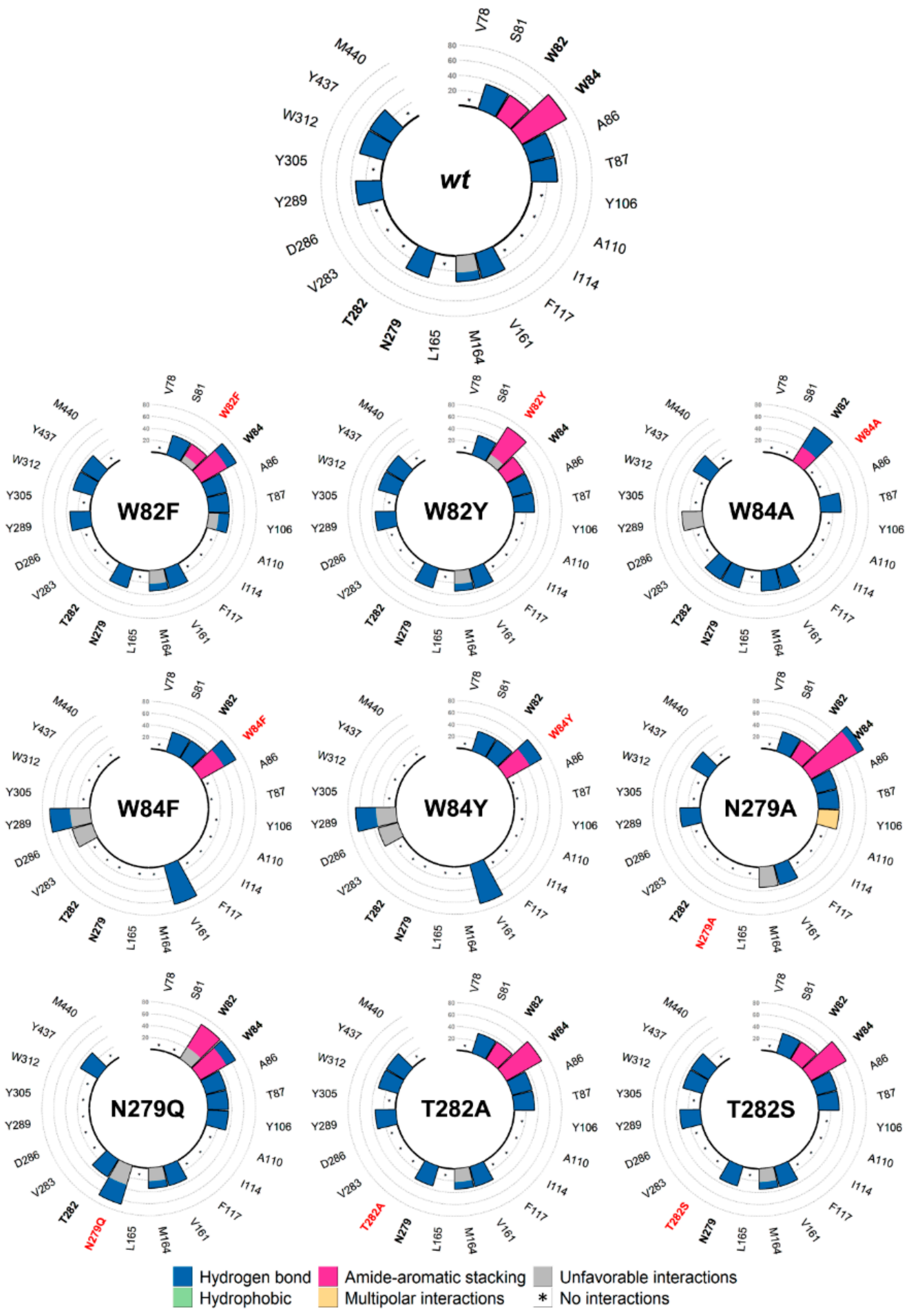

Figure S10. Interaction frequency between urea and binding site residues for each mutation assessed.

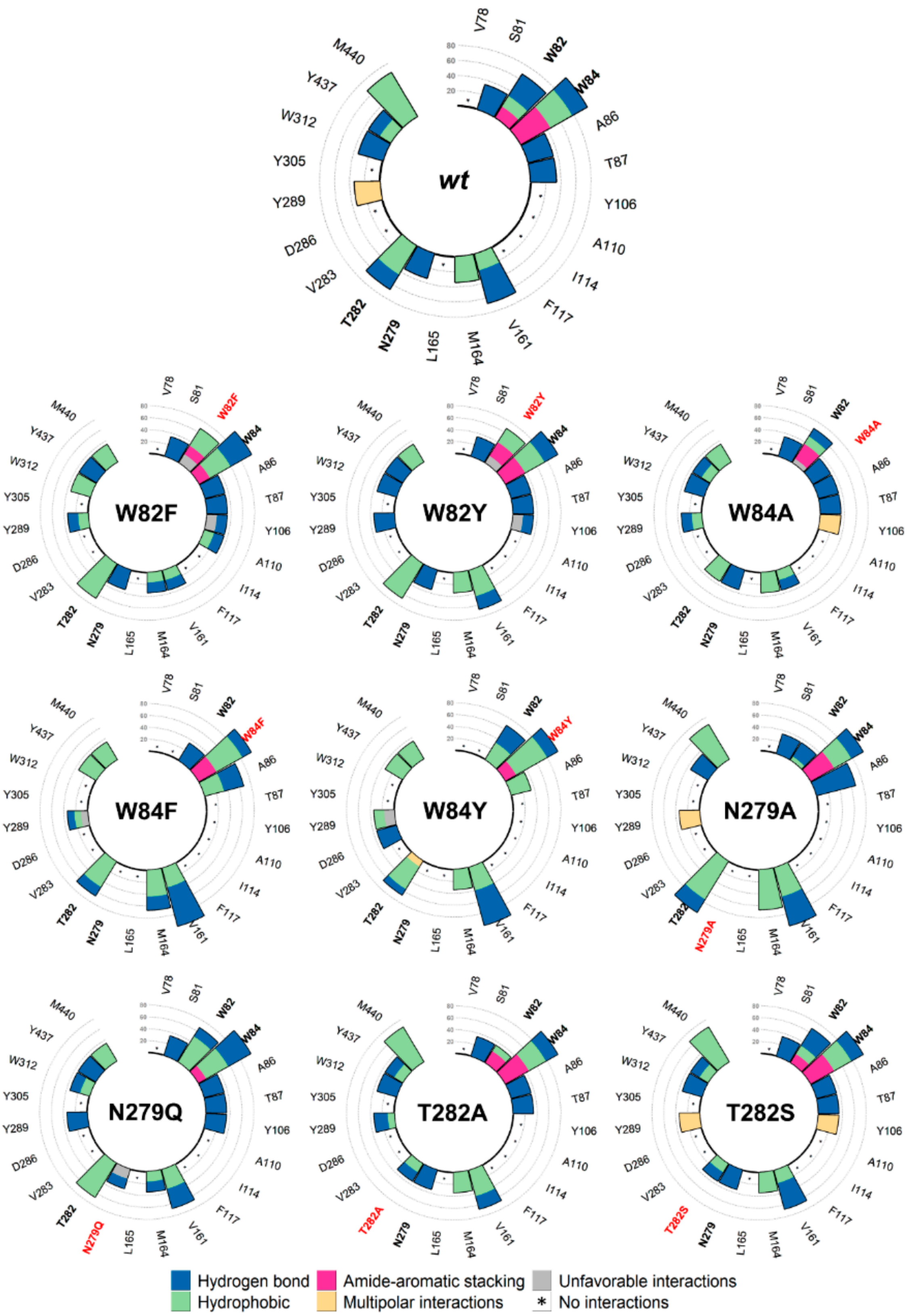

Figure S11. Interaction frequency between ACM and binding site residues for each mutation assessed.

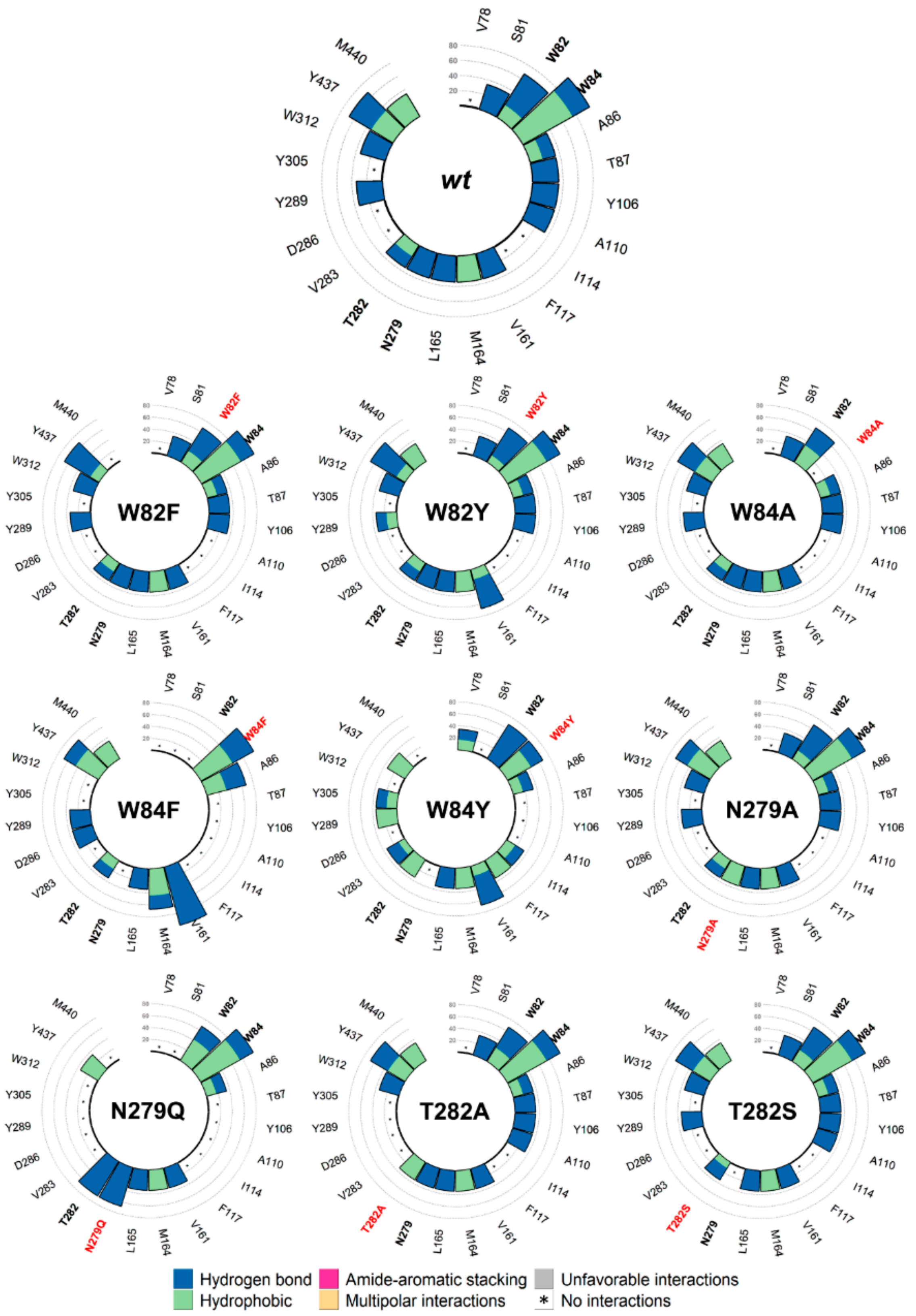

Figure S12. Interaction frequency between thiourea (2-TU) and binding site residues for each mutation assessed.

Table S1. *Aspergillus nidulans* strains used and constructed during this study.

Strain	*Genotype*
**MVD 001**	*pabaA1 veA1*
**P002**	*yA2 pabaA1*
**MVD 10A**	*ureA::gfp::AFpyrG riboB2 pyrG89 pyroA4 ΔnkuA::argB veA1*
**MVD 13A**	*ureAΔ::riboB riboB2 pyrG89 pyroA4 ΔnkuA::argB veA1*
**MVD 14A**	*yA2 ureAΔ::riboB riboB2 pyrG89 pyroA4 ΔnkuA::argB veA1*
**W82A**	*yA2 ureAW82A::gfp::AFpyrG pyrG89 pyroA4 riboB2 nkuA::argB veA1*
**W82F**	*yA2 ureAW82F::gfp::AFpyrG pyrG89 pyroA4 riboB2 nkuA::argB veA1*
**W82Y***	*yA2 ureAW82Y::gfp::AFpyrG pyrG89 pyroA4 riboB2 nkuA::argB veA1*
**W84A***	*ureAW84A::gfp::AFpyrG pyrG89 pyroA4 riboB2 nkuA::argB veA1*
**W84F***	*yA2 ureAW84F::gfp::AFpyrG pyrG89 pyroA4 riboB2 nkuA::argB veA1*
**W84Y***	*ureAW84Y::gfp::AfpyrG pyrG89 pyroA4 riboB2 nkuA::argB veA1*
**N279A***	*ureAN279A::gfp::AFpyrG pyrG89 pyroA4 riboB2 nkuA::argB veA1*
**N279Q***	*ureAN279Q::gfp::AFpyrG pyrG89 pyroA4 riboB2 nkuA::argB veA1*
**T282A***	*ureAT282A::gfp::AFpyrG pyrG89 pyroA4 riboB2 nkuA::argB veA1*
**T282S***	*ureAT282S::gfp::AFpyrG pyrG89 pyroA4 riboB2 nkuA::argB veA1*

* Obtained in this study.

Table S2. Oligonucleotides used in this study. Mutated codons are underlined. Modified bases are denoted in black.

Primer name	*5´to 3´Sequence*
**Ure5-F**	GAAACCTGGAGCAGTCGAAG
**Ure3-R**	CCCGATTTCTGAGACAAGGA
**Ure5-N**	GCACCGATGACAAGGGAGAT
**Ure3-N**	ACCAATGGATCTGGCACTAAAC
**W82Y-F**	TCGTGAGCAGT**TAC**ACCTGGGCAGCTACTCTGCTG
**W82Y-R**	TAGCTGCCCAGGT**GTA**ACTGCTCACGACAGCAGAG
**W84A-F**	AGCAGTTGGACC**GCT**GCAGCTACTCTGCTGCAATC
**W84A-R**	CAGAGTAGCTGC**AGC**GGTCCAACTGCTCACGACAG
**W84F-F**	AGCAGTTGGACC**TTC**GCAGCTACTCTGCTGCAATC
**W84F-R**	CAGAGTAGCTGC**GAA**GGTCCAACTGCTCACGACAG
**W84Y-F**	AGCAGTTGGACC**TAC**GCAGCTACTCTGCTGCAATC
**W84Y-R**	CAGAGTAGCTGC**GTA**GGTCCAACTGCTCACGACAG
**N279A-F**	ACCTCGTCGGT**GCT**TTCGGCACTGTCTTCCTGGAC
**N279A-R**	CAGTGCCGAA**AGC**ACCGACGAGGTTGATGACCCAG
**N279Q-F**	ACCTCGTCGGT**CAG**TTCGGCACTGTCTTCCTGGAC
**N279Q-R**	CAGTGCCGAA**CTG**ACCGACGAGGTTGATGACCCAG
**T282A-F**	ACCTCGTCGGTAACTTCGGC**GCT**GTCTTCCTGGACAACGGCTAC
**T282A-R/ T282S-R**	GCCGAAGTTACCGACGAGGTTG
**T282S-F**	ACCTCGTCGGTAACTTCGGC**TCT**GTCTTCCTGGACAACGGCTAC
